# The testis and epididymis are productively infected by SIV and SHIV in juvenile macaques during the post-acute stage of infection

**DOI:** 10.1186/1742-4690-4-7

**Published:** 2007-01-31

**Authors:** Miranda Shehu-Xhilaga, Stephen Kent, Jane Batten, Sarah Ellis, Joel Van der Meulen, Moira O'Bryan, Paul U Cameron, Sharon R Lewin, Mark P Hedger

**Affiliations:** 1 Infectious Diseases Unit, Alfred Hospital, Prahran, Australia; 2 Department of Medicine, Monash University, Alfred Campus, Prahran, Australia; 3 Department of Microbiology, Melbourne University, Melbourne, Australia; 4 Monash Institute of Medical Research, Clayton, Australia; 5 Peter McCallum Institute, Melbourne, Australia

## Abstract

**Background:**

Little is known about the progression and pathogenesis of HIV-1 infection within the male genital tract (MGT), particularly during the early stages of infection.

**Results:**

To study HIV pathogenesis in the testis and epididymis, 12 juvenile monkeys (*Macacca nemestrina*, 4–4.5 years old) were infected with Simian Immunodeficiency Virus mac 251 (SIV_mac251_) (n = 6) or Simian/Human Immunodeficiency Virus (SHIV_mn229_) (n = 6). Testes and epididymides were collected and examined by light microscopy and electron microscopy, at weeks 11–13 (SHIV) and 23 (SIV) following infection. Differences were found in the maturation status of the MGT of the monkeys, ranging from prepubertal (lacking post-meiotic germ cells) to post-pubertal (having mature sperm in the epididymal duct). Variable levels of viral RNA were identified in the lymph node, epididymis and testis following infection with both SHIV_mn229 _and SIV_mac251_. Viral protein was detected via immunofluorescence histochemistry using specific antibodies to SIV (anti-gp41) and HIV-1 (capsid/p24) protein. SIV and SHIV infected macrophages, potentially dendritic cells and T cells in the testicular interstitial tissue were identified by co-localisation studies using antibodies to CD68, DC-SIGN, αβTCR. Infection of spermatogonia, but not more mature spermatogenic cells, was also observed. Leukocytic infiltrates were observed within the epididymal stroma of the infected animals.

**Conclusion:**

These data show that the testis and epididymis of juvenile macaques are a target for SIV and SHIV during the post-acute stage of infection and represent a potential model for studying HIV-1 pathogenesis and its effect on spermatogenesis and the MGT in general.

## Background

Sexual transmission remains the main route of HIV-1 infection. The semen of the HIV-1 infected individual contains free virion particles and HIV-1 infected cells that come from the prostate, seminal vesicles and the urethra. The precise role of the testis and the epididymis in viral shedding during acute HIV infection is not known [1,2]. However, the immunologically privileged status of the testis and the existence of the blood testis barrier (BTB) have led to the speculation that the human testis in particular may be a reservoir and a potential sanctuary site for HIV-1 infection [3,2].

The main target cells for HIV-1, macrophages and CD4^+ ^T cells, are found in the interstitial space of the testis, between the seminiferous tubules [4]. CD4^+ ^T cells in the testis of HIV-1 infected men are the major cells infected by the virus in this tissue according to at least one study [5]. Whether HIV-1 infected CD4^+ ^T cells and macrophages are able to freely migrate from the interstitial tissue through the basal lamina of the seminiferous tubules in the intact testis, remains unknown. However, in the setting of orchitis in HIV-1 infected patients with AIDS, infiltration of tubules by CD4^+ ^T cells has been described [4]. It is also unclear whether resident macrophages and CD4^+ ^T cells of the testis are a target for infection in the early stages of HIV-1 infection or whether this is a feature only of the late stages of the disease. In the epididymis, CD4^+ ^T cells, CD45^+ ^(panleukocyte marker) cells such as macrophages are the most likely population to be infected [6,7]. It is believed that HIV-1 infected CD4^+ ^T cells, macrophages and spermatogenic cells from the testis and epididymis are shed into the semen during the course of HIV-1 infection [8,5], thus contributing to viral transmission, though further evidence to support this opinion is needed.

Human testicular macrophages express CD4, CD45, CCR5, CXCR4 and DC-SIGN suggesting that macrophages in the testis may be infected by HIV-1 and that these macrophages may be a site of early viral localization and a potential HIV-1 reservoir [9,10]. Infection of testicular macrophages has been demonstrated in an early study in HIV-1 patients with AIDS and orchitis [4] and recently evidenced in a study using human testis explants from healthy donors infected *in vitro *with a dual tropic HIV-1 strain [10]. However, at which stage of the disease macrophages of the testis are targeted by HIV *in vivo *remains unclear. SIV-infected macrophages and T cells have been reported in the MGT in chronically infected Macaques in the late stages of the disease. However, in almost all cases, this infection was also associated with inflammatory lesions within the testis [11]. Viral protein and HIV-1 DNA have been found not only within the interstitial tissue but also inside the seminiferous tubules, in the Sertoli cell and the germ cells [12-14]. However much of the results obtained concern the late stage of the disease and remain controversial [15].

HIV-1 infection of the epididymis remains poorly defined. Using PCR in situ hybridization analysis, a study of the epididymides of HIV-1 infected men that died from AIDS has reported the presence of HIV-1 DNA within the connective tissue of the epididymis of 1 out of 8 cases [8]. In contrast, a different study detected HIV-1 positive cells of lymphocytic morphology within both the epididymal epithelium and the connective tissue stroma [4]. Extensive studies on the pathogenesis of HIV-1 infection in the epididymis are currently lacking.

In the present study, the testis and epidydimis was examined shortly following infection of macaques with SIV and SHIV. We report that SIV and SHIV infect the MGT with similar patterns. SIV and SHIV RNA are detectable in both the macaque testis and the epididymis in the post acute phase of infection. These viruses target testicular macrophages, T cells and spermatogenic cells in the early phases of infection.

## Results

### 1. Macaques of similar age and body weight display differences in their sexual maturation

**Figure 1:**
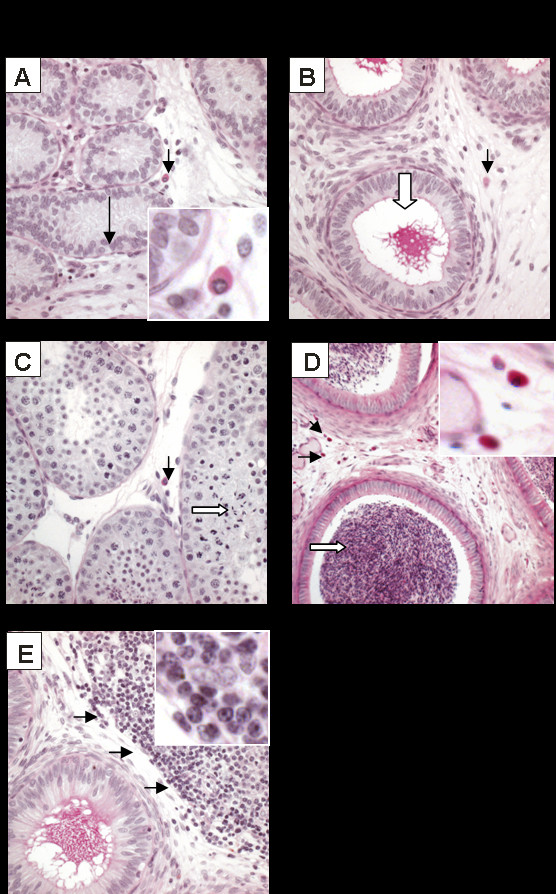
**Light microscopy of the macaque testis and epididymis (PAS staining)**. A. Testis of an immature monkey: only seminiferous cords containing Sertoli cells and basally situated spermatogonia (long arrow) are present. B. Epididymis of immature monkey: note absence of germ cells in the epididymal lumen (block arrow). C. Spermatogenesis occuring in the testis of a more mature monkey (block arrow indicates elongated spermatids). D. Sperm present in the epididymal lumen of a mature monkey (block arrow). E. Epididymides of some infected monkeys were characterized by the presence of large mononuclear cell infiltrates (arrows). Macrophages are present in the testicular and epididymal tissues (short arrows, panels A – D). Indicated macrophages and T cells are also shown in higher magnifications (panels A, D and E).

Out of 12 SIV/SHIV infected animals, five were either pre-pubertal (i.e. lacking meiotic germ cells) or showed only early signs of spermatogenesis (i.e. meiotic spermatocytes were the most mature germ cells in the testis) (Fig. 1, panels A and B). Developmental and infection data are summarised in Table 1 and detailed histologic observations have been summarized in Table 2. Six animals displayed more advanced stages of spermatogenesis characterized by the presence of mature late spermatids in the epithelium either with or without the presence of mature spermatozoa in the epididymal lumen (Fig. 1, panels C and D; Tables 1 and 2). In general, the numbers of macrophages and T cells throughout the testicular interstitium appeared to increase with increasingly progressive spermatogenesis, however, four of the prepubertal animals (two SIV infected and two SHIV infected macaques) had minor or significant testicular mononuclear cell infiltrates (Table 2). The epididymal interstitium (stroma) of seven of the animals (5 SIV infected monkeys, 2 SHIV infected monkeys) contained significant lymphocytic infiltrates (Fig. 1, panel E; Tables 1 and 2).

**Table 1:** Assessment of macaque development and other infection characteristics.

Animal nomenclature and viral strains	Body weight (kg)	Plasma RNAVLs Log 10/copies/ml	CD4 T cell counts (% of total peripheral lymphocytes)	Lymph node VLs (SIV RNA copies/20 ng RNA)	Testis VLs (SIV RNA copies/20 ng RNA)	Epididymis VLs (SIV RNA copies/20 ng RNA)	Maturation Status
M1_SIVmac251_	6.75	6.29	7.9	190	600	1150	+++
M2_SIVmac251_	6.50	6.25	8.1	65 × 10^4^	110	2200	-
M3_SIVmac251_	5.60	5.97	26.3	260	<50	<50	+
M4_SIVmac251_	6.25	7.33	32.5	<50	385	<50	++
M5_SIVmac251_	5.30	6.06	17.4	<50	75	<50	-
M6_SIVmac251_	7.20	4.40	30.0	<50	<50	<50	+++
M7_SHIVmn229_	7.45	5.48	1.1	1 × 10^3^	4000	125	+++
M8_SHIVmn229_	5.80	4.82	1.5	125 × 10^2^	55	<50	-
M9_SHIVmn229_	4.30	6.52	0.4	15 × 10^2^	90	80	-
M10_SHIVmn229_	5.50	4.77	1.4	13 × 10^3^	<50	65	++
M11_SHIVmn229_	7.35	5.85	0.4	42 × 10^2^	<50	150	-
M12_SHIVmn229_	4.80	4.76	0.3	85 × 10^3^	<50	<50	++

- animal is prepubertal (no active spermatogenesis)

+ animal is early pubertal (early meiotic cells only in the seminiferous epithelium)

++ animal is late pubertal (elongating spermatids in seminiferous tubules, but no sperm in epididymis)

+++ animal is sexually mature (full spermatogensis in the seminiferous tubules and sperm in the epididymal lumen)

VL-viral load; TCID50 = 50% Tissue Culture Infective Dose.

**Table 2:** Summary of testicular and epididymal histology for SIV and SHIV-infected macaques

**Animal**	**Spermatogenesis**	**Testicular Interstitium**	**Epididymis**
M1_SIVmac251_	Full spermatogenesis	Normal macrophage numbers; no infiltrates	Sperm in lumen; macrophages and small sized mononuclear cell infiltrates in stroma
M2_SIVmac251_	Spermatocytes and seminiferous cords only	Small numbers of scattered mononuclear cells; macrophage numbers appear normal	No cells in lumen; large sized mononuclear cell infiltrates in stroma; normal macrophage numbers
M3_SIVmac251_	Some round spermatids present	Few macrophages; no infiltrates	No cells in lumen; numerous macrophages and focal mononuclear cell infiltrates
M4_SIVmac251_	Elongating spermatids present; some tubules with lumen	Normal macrophage numbers; no infiltrates	Sperm and many spermatocytes and round spermatids in lumen; several very large mononuclear cell infiltrates in stroma
M5_SIVmac251_	Spermatocytes and seminiferous cords only	Minor mononuclear cell infiltrates; macrophage numbers appear normal	No cells in lumen; medium sized mononuclear cell infiltrates in stroma; normal macrophage numbers
M6_SIVmac251_	Full spermatogenesis	Normal macrophage numbers; no infiltrates	Sperm in lumen; no stromal infiltrates
M7_SHIVmn229_	Full spermatogenesis	Normal macrophage numbers; no infiltrates	Sperm in lumen; numerous macrophages and scattered mononuclear cell infiltrates
M8_SHIVmn229_	Spermatogonia and seminiferous cords only	Few macrophages; no infiltrates	No cells in lumen; no stromal infiltrates
M9_SHIVmn229_	Spermatogonia and seminiferous cords only	Few macrophages; no infiltrates	No cells in lumen; no stromal infiltrates
M10_SHIVmn229_	Elongating spermatids present; some tubules with lumen	Normal macrophage numbers; no infiltrates	No cells in lumen; numerous macrophages and scattered mononuclear cell infiltrates
M11_SHIVmn229_	Spermatogonia and seminiferous cords only	Numerous macrophages in the interstitium and some focal mononuclear cell infiltrates	No cells in lumen; no stromal infiltrates
M12_SHIVmn229_	Elongating spermatids present; spermatogenesis is disorganised	Normal macrophage numbers; no infiltrates	No sperm in lumen; degenerating spermatocytes and round spermatids in lumen; no stromal infiltrates

### 2. Dendritic cells are present in the interstitium of the macaques testis

**Figure 2:**
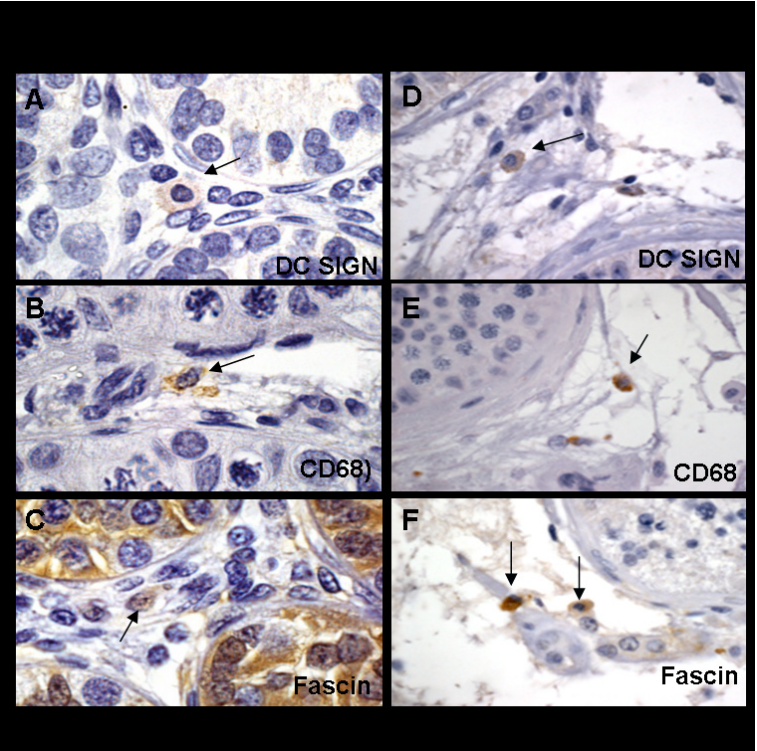
**Immunohistochemistry of SIV/SHIV-infected pre-pubertal macaque testis (A-C) and normal adult human testis (D-F)**. Human testis tissue was used as positive control for antibodies targeting immune cells present in the testis. Formalin-fixed, paraffin-embedded testis tissue was stained with isotype matched control (IgG_1_, data not shown), a macrophage and dendritic cell marker (DC-SIGN, panel A and D), a myeloid cell marker (CD68, panel B and E) and a dendritic cell marker (Fascin/p55, panels C and F). DAB-positive cells indicated by arrows in sections conterstained with haematoxylin.

Staining of testicular tissue with the DC-SIGN antibody established the presence of DC-SIGN^+ ^cells in the testicular interstitium (Fig. 2, panel A and D). CD68^+ ^cells were observed in both the monkey and human testis, indicative of the presence of macrophages (Fig. 2, panel B and E). Because DC-SIGN is a DC and a macrophage marker we probing our sections with an antibody to Fascin, a 55 kDa protein involved in antigen presentation [16] that is present only in mature DCs [17]. Probing with this antibody confirmed the presence of a DC population in this organ (Fig. 2, panel C and F).

### 3. Testis and epididymis are infected by SIV and SHIV-1 infection upon the establishment of viremia

**Figure 3:**
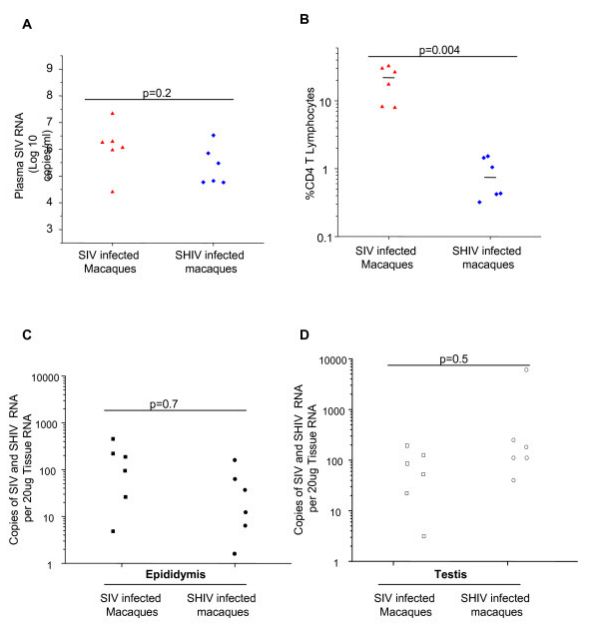
**Plasma and tissue viral RNA levels in SIV and SHIV-infected macaques**. Both animals groups displayed similar plasma viral loads at the time of tissue collection (p = 0.2)(panel A). The percentage of CD4^+ ^T lymphocytes in the peripheral blood of SIV infected macaques at the time of tissue collection was significantly higher than in SHIV infected macaques (p = 0.004) (panel B). Epididymal (panel C) and testicular (panel D) SIV RNA viral loads were also similar between SIV and SHIV infected macaques.

All animals reached the peak of infection two weeks post-challenge (data not shown). Plasma SIV RNA viral loads were, however, similar in both macaque groups at time of termination and tissue collection (Fig. 3A). In SHIV infected macaques, viral loads were associated with a greater diminished percentage of CD4 T cells compared to the CD4 T cell depletion observed in SIV infected animals (Fig. 3B). SIV RNA was detected in the epididymis and testis (Fig. 3, panels C and D), and lymph node tissues (Table 1). While there was a statistically significant difference between viral loads in the lymph nodes of the two groups (p = 0.05) (Table 1), there was no difference between SIV and SHIV infected animals, nor the immature and mature macaques with respect to viral RNA levels in the testis and epididymis (p > 0.08) (Fig. 3, panels C and D). The range of values, however, was very large and examination of tissue from a larger cohort of infected animals may clarify whether a real difference exists between the MGT tissue values.

### 4. Viral infection potentially leads to abnormal spermatogenesis in the testis and epididymis

**Figure 4:**
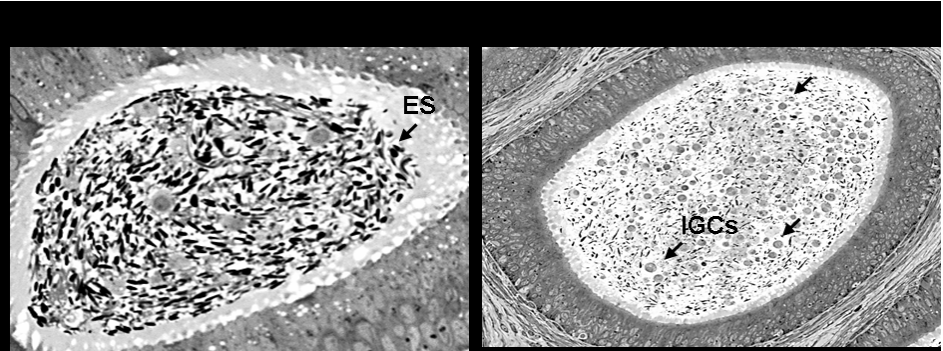
**Electron microscopy analysis of the epididymis of infected macaques (x100)**. Premature sloughing of immature germ cells (spermatocytes and spermatids, arrows) into the epididymal lumen of some pre-pubertal SIV and SHIV infected macaques (panel B), a pattern that was distinct from that of the epididymal lumen of other mature macaques (panel A). **ES: **elongated spermatids; **IGCs**: immature germ cells.

Despite detectable SIV RNA in the testis and epididymis, structures resembling retroviral particles were rarely observed via EM (data not shown). This is not surprising as detection of viral particles in tissues is generally difficult. Samples submitted for EM were additionally analysed for their content of the epididymal tubules (Fig. 4). Interestingly, in one SIV infected (M4) and one SHIV infected late pubertal macaque (M12) we observed round cells resembling immature germ cells being shed into the epididymal lumen (Fig. 4B).

### 5. SIV targeted immune cells of the testis and epididymis and the developing germ cells

**Figure 5:**
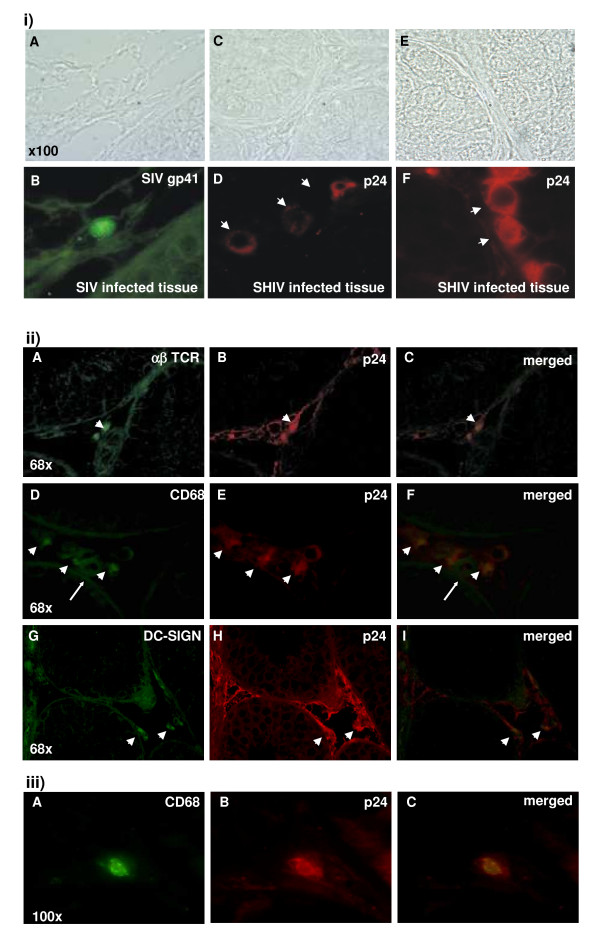
**Identification of SIV and SHIV target cells in the testis and epididymis of infected macaques**. (i) Gp41 SIV positive cells in the interstitium of the testis (panels A and B, phase-contrast micrograph of section under bright-field versus immunofluorescence micrograph of same area, respectively). P24 Gag (HIV) or SIV/SHIV capsid positive cells in the testis interstitium (arrow heads) and seminiferous tubules of a pre-pubertal animal (long arrow) (panels C and D). Strong p24 positive staining of spermatogonia in SHIV infected macaque (panels E and F). The frequency of infected cells in SIV infected animals (counted 5 high magnification fields/section, bright field versus stained nr of cells) is from 1–5 positive cells/tubule (out of 16–20 total number of cells) and up to 50 % of total germ cells infected in some of the SHIV infected macaques (6 cells infected in one tubule in the example shown). (ii) Individual staining and merged images of a αβTCR^+^/p24^+ ^double positive cell in the testicular interstitium of a pubertal macaque (panels A, B and C). CD68^+^/p24^+ ^double positive cell in the testicular interstitium of a pre-pubertal macaque (panel D, E and F). A representative image of two DC-SIGN^+^/p24^+ ^double positive cells in the testicular interstitium of a pre-pubertal macaque (panels G, H and I). (iii) CD68^+^/p24^+ ^double positive cell in the epididymis of a pubertal macaque (panels A, B and C).

IF staining with antibody to SIV gp41 showed the presence of infected cells in the interstitial space of four out of six, mainly pre-pubertal SIV-infected macaques (Fig. 5i, panel B). However, because of the staining pattern of this antibody (punctuate, with a fuzzy staining of the whole cell cytoplasm), subsequent double staining procedures were performed using an antibody to HIV-1 p24 capsid protein that crossreacts with SIV p27. We observed positive staining for capsid protein in the testis interstitium and the seminiferous tubules of all SIV-infected macaques (Fig. 5i, panel D. Strong single positive staining with anti-p24 antibody of basally-located cells inside the seminiferous tubules was indicative of productive infection of spermatogonia (Fig. 5i, panel F). Co-localization of positively stained αβ TCR^+ ^cells with p24 in the testis was indicative of infection of T cells (Fig. 5ii, panels A-C). Co-localization of positively stained CD68^+ ^cells with p24 in this organ was indicative of infection of cells of the myeloid lineage in mature and immature animals (Fig. 5ii, panels D-F). Co-localization of positively stained DC-SIGN^+ ^cells with p24 in this organ was indicative of infection of macrophages and potentially DCs in SIV and SHIV infected, mature and immature macaques (Fig. 5ii, panels G-I). CD68^+^/p24^+ ^cells were also detected in the epididymal tissues of SIV and SHIV infected macaques (Fig. 5iii, panels A-C).

## Discussion

In this study, we have used SIV and SHIV infected macaques to determine the characteristics of post-acute SIV and SHIV infection in the testis and epididymis. Our data demonstrate (i) significant SIV RNA viral load in both testis and epididymis upon establishment of viremia, (ii) productive infection of immune cells of the testis and epididymis, (iii) SIV and SHIV infection of spermatogonia, but not the later germ cells, (iv) the presence of a DC population in the monkey testis and (v) the presence of immature testicular germ cells (spermatocytes and round spermatids) in the epididymal lumen, which may be related to the infection. Although the animal cohort was relatively small in size and the animal maturation status differed between animals of both groups, this study clearly demonstrates that SIV and SHIV infection of the MGT is an important site of viral replication during SIV and SHIV infection. Based in the VLs observed in the testis and epididymis we conclude that these two organs do not significantly contribute to the overall viremia. However, because free virions and infected cells are shed from these organs into semen, the observed VLs are potentially very significant in terms of male to female viral transmission.

With disease progression, HIV-1 infection disrupts the testis morphology and spermatogenesis, reviewed in [2]. Our data show potentially significant levels of viral RNA within the testis tissue even within 11–13 weeks and 23 weeks of infection for SHIV-infected versus SIV infected macaques, well prior to the development of simian AIDS. At week 23, despite high SIV viral loads, the percentage of CD4 T-cells was relatively preserved for SIV infected macaques but was substantially reduced in SHIV infected macaques. Moreover, infected immune cells were observed in the testis interstitium and epididymal stroma. Thus, infection of both testicular and epididymal cells could potentially act as a reservoir for semen, throughout the duration of infection [18], reviewed in [2].

In acute infection, plasma viral loads correlate with viral loads in semen, suggesting that the MGT (prostate, seminal vesicles, urethra, testis, epididymis) may be targeted by the virus in the very early stage of infection [19]. While seminal plasma and seminal leukocytes originate mainly from the infected prostate and seminal vesicles, most of the cells present in semen are germ cells originating from the testis and epididymis. Macrophages and T cells in the testis of the infected macaques were infected. It is possible that these infected cells are passed onto the epididymis and contribute to viral shedding in semen early in infection. This might occur at the rete testis where translocation of T cells and macrophages into the lumen of the duct can occur [20-22]. There is also evidence that macrophages and T cells found in the epididymal epithelium may also cross into the epididymal duct [23,24]. It is however important to note that we have shown no evidence that infected T cells or macrophages move from the testis or epididymal tissue into the duct. The more rapid progression of SHIV_mn229 _compared to SIV infection of macaques was consistent with previous reports [25,26]. As expected, plasma and lymph node viral load was significantly higher in SHIV infection when compared with SIV infection, but there was no significant difference in viral load in the testis and epidydimis from SHIV infected animals compared with SIV-infected animals. This possibly suggests that infection of the MGT occurs relatively early following infection and is not dependent on disease progression, the degree of immunosuppression of the host or co-receptor usage.

Infection of macrophages in the MGT with SIV and SHIV was infrequent. Most DC-SIGN^+ ^cells were negative for p24 staining in the SHIV challenged monkeys. Similarly, a low number of infected macrophages has been demonstrated in the female genital tract via *in situ *hybridization [11]. Using the more specific method of double IF, our data reinforce these findings. We were unable to comment on MGT cell loss (i.e. T cell loss due to infection versus other maturation related cell numbers) due to SIV and SHIV infection as we did not have access to serial biopsies or maturation matched tissues from naive animals. Although DCs have been observed previously in the rodent testis [22]and very recently in the human testis [10], the identification of a dendritic cell population in macaque testis is a novel finding.

There have been very few studies of the non-human primate reproductive tract during the pre-pubertal period [27]. In the course of this study, in at least two animals infected with SHIV and one SIV infected animal, we have interestingly observed the presence of spermatocytes and round and elongated spermatids in the epididymal lumen. Autofluorescence of spermatocytes, spermatids and mature sperm made it difficult to determine whether these cells are infected in the epididymis of SIV and SHIV macaques. These round and elongated spermatids did not stain with the antibody to p24, suggesting that they are uninfected. We are in the process of establishing a technique that will specifically determine the presence of SIV and SHIV RNA and/or DNA in these cells. Whether their presence in the epididymal lumen is a consequence of the viral infection of this organ or simply a normal maturation process in non-human primate sperm maturation biology remains to be determined. The spermatogonia of these animals were positive for p24 antigen, suggesting ongoing productive infection within the seminiferous tubules. This would lead to impaired spermatogenesis even in early HIV infection.

Infection of spermatogonia has only been previously reported by one study [28], though not via IF. Meiotic and post-meiotic germ cells could potentially get infected by HIV-1 due to clonal infection (i.e. from one infected germ cell to the next) [28]. Thus, HIV-1 DNA has been detected within different categories of more mature germ cells, possibly through clonal infection. We saw no evidence of later germ cells being productively infected suggesting that either infected spermatogonia die rather than develop further, a conclusion that supports the previously described arrest of spermatogenesis in men that have died from AIDS [29], or later germ cells are latently infected and do not support productive infection. Spermatogonia would represent the initial target for HIV-1 infection. The spermatogonia are located outside the Sertoli cell tight junctions, making them readily accessible to virions present in the interstitium. While the presence of the galactosylceramide receptor has been reported on their surface [30], the presence of other HIV receptors on spermatogonia remains unclear. Two studies have reported the presence of viral RNA or proteins within germ cells [31,8]. We detected productive infection in spermatogonia located in seminiferous tubules using IF. A productive infection would lead to the release of free HIV-1 particles into the seminiferous tubules lumen and subsequently in the seminal fluid. It is not clear how infection of these cells is initiated, although one possibility would be virus transcytosis through the blood testis barrier, a phenomenon that is yet to be observed.

## Conclusion

In summary, we provide evidence that SIV/SHIV infects the testis and epididymis of macaques during early infection. We also observed abnormalities in the morphology the testis and epididymis following SIV/SHIV infection that may be related to the infection. These data suggest infection of the MGT may be a significant viral reservoir for semen infection that is established early following infection with potential consequences on MGT function.

## Materials and methods

### Animals

Colony-bred simian retrovirus type D-negative pigtail macaques (*Macacca nemestrina*, aged approximately 4–5 years) were anesthetized with Ketamine (10 mg/kg of body weight, intramuscularly) prior to all procedures. All procedures and protocols were approved by the University of Melbourne Animal Experimentation Ethics Committee in accordance with the guidelines of the National Health and Medical Research Council of Australia (NHMRC).

### SIV/SHIV infection of macaques

Macaques were inoculated with either (i) SIV_mac251 _(n = 6), a CCR5-utilising strain that results in gradual depletion of CD4 T cells (10^6 ^50% tissue culture infective doses [TCID_50_], intravenously) [32], or (ii) SHIV_mn229 _(n = 6), a virulent mucosal CXCR4-utilizing HIV-1_IIIB_-based strain which results in rapid CD4 T cell depletion, in two 0.5-ml doses over 2 days (total 10^5 ^TCID_50_, intrarectally) [25,33,26]. Testis and epididymal tissue from SIV-infected macaques were collected at week 23 post-infection and those of SHIV-infected macaques were collected between weeks 11–13 post infection.

### Tissue collection and processing

Inguinal lymph nodes, testis and epididymis were surgically removed immediately after euthanasia. Tissues were then dissected to 1 mm size fragments and (i) snap frozen in liquid nitrogen for RNA isolation, (ii) fixed for EM analysis, (iii) stored in Bouin's fixative for preparation of tissue slides and structural analysis or (iv) frozen in OCT for immunohistochemistry (IHC).

### RNA isolation, reverse transcription and real-time PCR analysis

RNA was prepared using the Trizol method following the manufacturer's instructions (Invitrogen, CA, USA). Approximately 20 ng of RNA of each tissue was used in the reverse transcription (RT) reaction. Subsequently, the entire cDNA obtained by reverse transcription was used to determine tissue viral loads in a real-time PCR reaction. SIV and SHIV tissue viral loads and viremia were quantified by reverse-transcriptase real-time PCR, as previously described [34]. Briefly, SIV-GAGF01 5'-AATTAGATAGATTTGGATTAGCAGAAAGC and SIV-GAGR02 5'-CACCAGATGACGCAGACAGTATTAT were used as forward and reverse primers and the MGB TaqMan probe SIV-GAG 6FAM-CAACAGGCTCAGAAAA-MGBNFQ. The PCR was performed using freshly prepared cDNA on the Prism 7700 sequence detection system PCR thermal cycler (ABI) under the following conditions: 95°C 10 min followed by 45 cycles of 94°C 15 s and 61°C 60 s. GAPDH was quantified by using real-time PCR and a molecular beacon, as previously described [35], where hexachlorofluorescein (HEX) served as the reporter fluorochrome. The lowest limit of detection of the assay was 40 copies/ml. The VL data per each animal is expressed as number of viral RNA copies/20 ng of total tissue RNA.

### Flow cytometry analysis

The percentage of CD4^+ ^T cell population in Macaques blood was determined via flow cytometry, as previously described [25,33].

### Light microscopy (LM)

Testis, epididymis and lymph node sections were fixed overnight in Bouin's fluid and processed through standard paraffin-embedding techniques. 5 μM tissue sections were stained using either haematoxylin and eosin or periodic acid Schiff (PAS) and haematoxylin. Sections were examined on a light microscope (Leitz, Wetzlar, Germany). Photographs were taken using a digital camera (Leica MPS 60, Vienna, Austria) attached to a light and fluorescent microscope (Leica DMR).

### Thin section electron microscopy (EM)

Tissue biopsies were washed with 0.1 M sodium cacodylate buffer plus 5% sucrose and fixed in a 0.1 M sodium cacodylate solution containing 2% paraformaldehyde and 2.5% glutaraldehyde. Following washing in 0.1 M sodium cacodylate buffer containing 5% sucrose, tissue samples were post-fixed in 1% osmium tetroxide in 0.1 M sodium cacodylate buffer. Samples were then washed in distilled water, dehydrated through a graded series of alcohols before embedding in Spurrs Resin according to standard electron microscopy protocol. Ultrathin sections were cut with a diamond knife (Diatome, Vienna, Austria) using a ultra-microtome (Leica Ultracut S), stained with both methanolic uranyl acetate and lead citrate before viewing in a transmission electron microscope (JEOL 1011, Tokyo, Japan) at 60 kV. Images were recorded with a MegaView III CCD cooled digital camera (Soft Imaging Systems, Münster, Germany).

### Immunohistochemistry (IHC)

Phenotyping of testicular and epididymal immune cells was performed using the Dako Autostainer kit (Dako, Carpinteria, CA), as described previously [25]. Normal, uninfected testis tissue used as negative controls was obtained with consent from a post-pubertal healthy donor with unexplained testicular pain requiring orchidectomy. The human testis tissue was used as a positive control for the antibodies used to identify immune cell populations in the macaque testis. Collection and analysis of human tissue was approved by the Monash Medical Centre Human Ethics Committee and was consistent with the appropriate NHMRC guidelines. Bouin's fixed testes, epididymides and lymph nodes were processed into paraffin, as previously described [36]. Paraffin-embedded tissue sections were dewaxed and rehydrated. To improve the sensitivity of the antibodies, all sections were subjected to an antigen retrieval step (7 min high/7 min low energy microwave (800 W), sodium citrate, pH 6.0). Endogenous peroxidase was blocked by incubation in Peroxidase Block (Dako), as previously described [36]. Sections were subsequently incubated anti CD-68 (PG-M1, Dako) for the detection of cells of the myeloid lineage, anti DC-SIGN (DC28, National Institutes of Health Reagent Repository) and anti-Fascin mouse monoclonal antibodies (Dako) for the detection of macrophages and dendritic cells (10 μg/ml) for 2 hrs, followed by an Envision polymer-mouse-horseradish peroxidase (Dako) for 15 min and visualized using diaminobenzidine tetrahydrochloride (Dako). Sections were counterstained using Mayer's hematoxylin and mounted under glass using p-xylene-bis (N-pyridinium bromide) and were analyzed and photographed (BH2 microscope; Olympus, Tokyo, Japan).

### Immunofluorescence (IF)

The specific distribution of SIV and SHIV protein was determined using a primary mouse monoclonal anti-gp41 antibody (clone KK15) raised against the envelope of SIV (1:100) and a mouse monoclonal antibody against p24 HIV-1 protein that cross reacts with SIV Gag (clone 91-6) (1:100) in IHC analysis and western blotting (data not shown). Both antibodies were obtained from the National Institutes of Health Repository, Bethesda, MD, USA. Following probing with primary antibodies, sections were stained with anti-mouse Alexa 488 (Green) and Alexa 596 (Red) conjugated secondary antibodies. A double staining IF protocol was used to determine the phenotype of infected cells in both tissues with antibodies raised in the same species, as previously described [37]. To identify the cellular location of SIV and SHIV proteins, antibodies used for phenotyping in the immunohistochemical analysis [anti-CD68, anti DC-SIGN, anti-αβT cell receptor (TCR) mouse anti-rat monoclonal antibody] were also used for IF. The TCR αβ mouse anti-rat monoclonal antibody was a kind gift of Dr. Nikolik-Paterson, Dept. Nephrology, Monash Medical Centre, Australia). Isotype controls were used for all antibodies employed in this study.

### Statistical analysis

Statistical analysis was performed using SPSS version 13.0 for Windows student version (LEAD Technologies, Inc.). Comparisons of clinical differences between patient groups were analyzed using the Mann-Whitney U-test. Correlations were examined using Spearman's rho test for non-parametric values.

## Competing interests

No financial competing interests are declared.

## Authors' contributions

MSX designed the experiments, performed RNA extractions for viral loads, IHC and IF studies and wrote the manuscript; MH analysed light microscopy data, provided senior expert advise and revised the manuscript, SK and JB provided the tissues, JB performed real-time PCR, SE performed the EM analysis, JVM helped with IF and preparing the figures, MOB provided the human testis tissue, PC and SRL provided expert advice and revised the manuscript.
